# A Rare Case of Cardiac Echinococcosis: The Role of Multimodality Imaging

**DOI:** 10.1016/j.case.2021.03.002

**Published:** 2021-04-28

**Authors:** Johan O. Wedin, Rafael M. Astudillo, Siri Kurland, Karl-Henrik Grinnemo, Rafael Astudillo, Per Vikholm, Petter Schiller

**Affiliations:** a Department of Cardiothoracic Surgery, Uppsala University Hospital, Uppsala, Sweden; b Department of Surgery, Västerås County Hospital, Västerås, Sweden; c Department of Infectious Diseases, Uppsala University Hospital, Uppsala, Sweden

**Keywords:** Echinococcus, Global longitudinal strain, Hydatid cyst, Open heart surgery, Transthoracic echocardiography

## Abstract

• Multimodality imaging plays a crucial role in confirming cardiac echinococcosis.
• Speckle-tracking echocardiography gives information about regional myocardial function.
• Cyst rupture often causes life-threatening anaphylactic shock.
• We present an unusual case of cyst rupture, with chest pain as the only symptom.
• Complete surgical removal is the treatment of choice for cardiac echinococcosis.

## Graphical abstract

**Figure:**
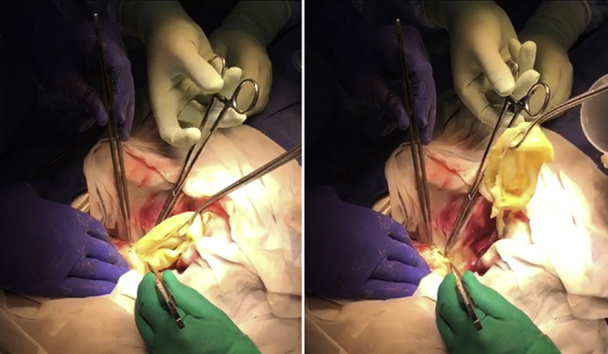

## Introduction

Echinococcosis is a parasitic tapeworm infection caused by the *Echinococcus* genus. Human echinococcus infection is very rare in high-income countries, while the incidence in some endemic areas might exceed 10%, making it a major health issue and economic burden.^1^ All internal organs can be affected in what is known as cystic echinococcosis, or hydatidosis, with *E. granulosus* being the most common causative pathogen.^2^ Cardiac echinococcosis is rare, and infected individuals may be asymptomatic for years due to the long incubation time.^3^ The diagnosis is often established through a combination of echocardiography, computed tomography (CT), and cardiac magnetic resonance imaging (cMRI). The use of speckle-tracking echocardiography has not been reported in the setting of cardiac echinococcosis but could provide detailed, objective, and reproducible information about regional cardiac function. Due to the risk of cyst rupture, and subsequent potential fatal anaphylactic shock, prompt surgical excision through open-heart surgery is recommended.^4^

## Case Presentation

**Figure 1:**
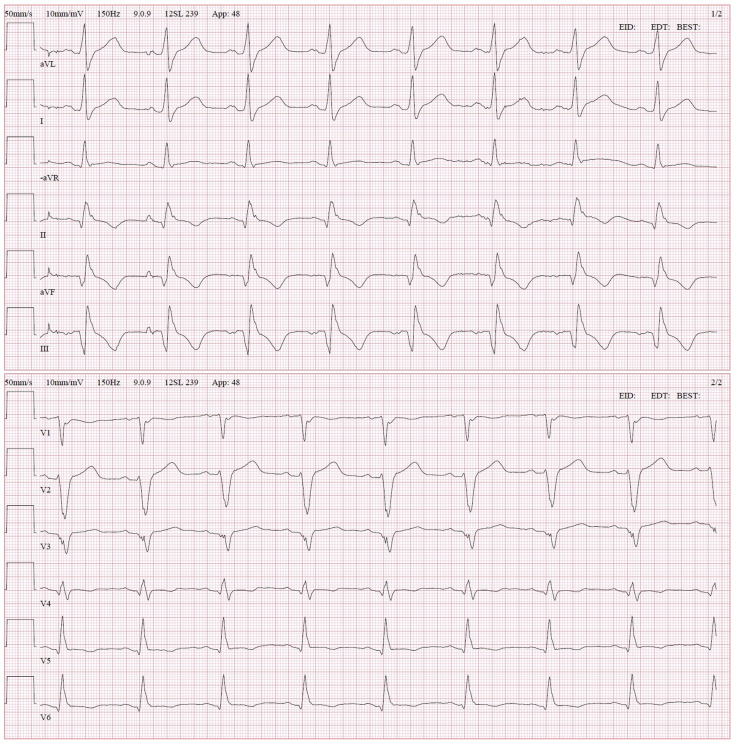
The electrocardiogram at admission to our clinic showed pathological Q waves and negative T waves in inferior leads (II, aVF, and III), negative T waves in lateral leads (V4-V6), and a poor R-wave progression in anterior leads.

**Figure 2:**
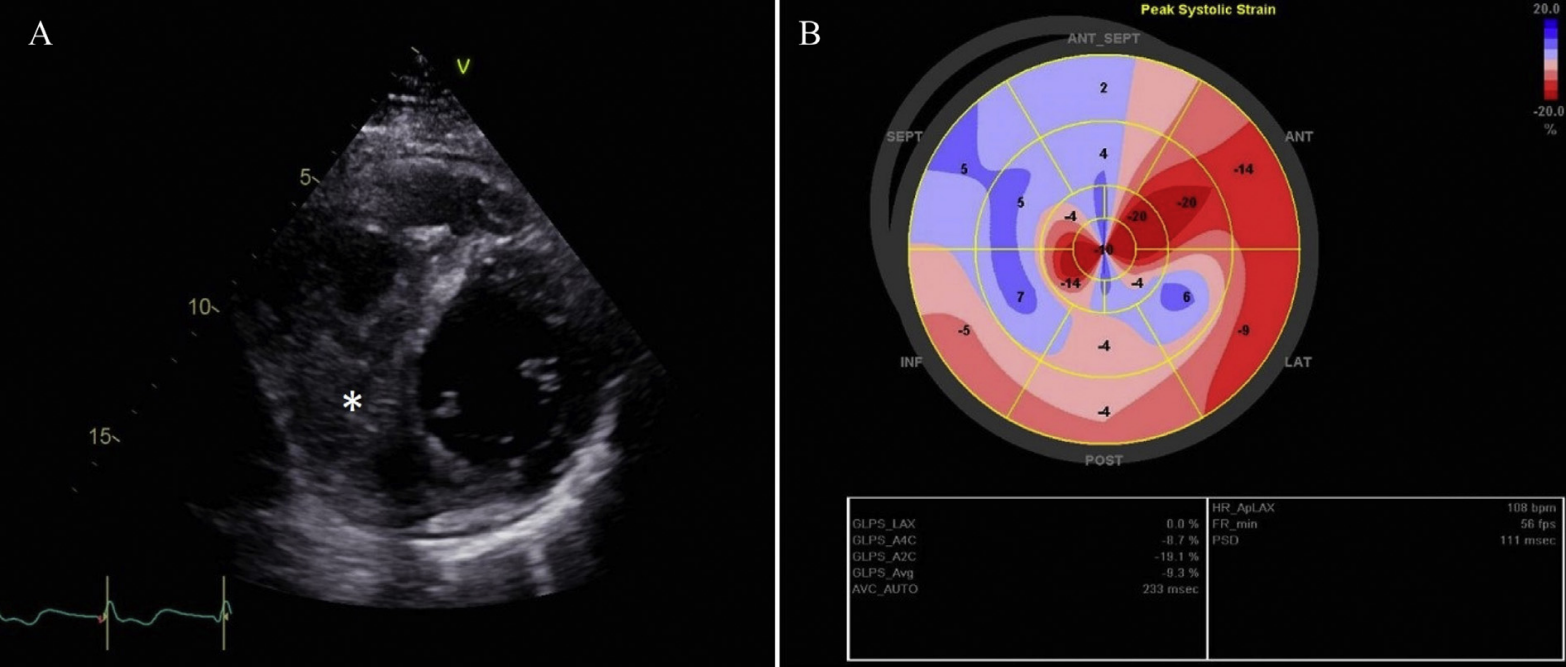
Transthoracic echocardiogram was initially performed due to suspected myocarditis. It revealed a large mass with hypoechogenic areas and a mildly to moderately reduced LVEF (40%) due to hypo- and dyskinesia in the affected areas. **(A)** The parasternal short-axis view at the papillary muscle level showing the mass in the inferoseptal wall and a minimal pericardial effusion. **(B)** The bull's-eye plot from the two-dimensional speckle-tracking echocardiography analysis visualizing the regional wall motion abnormalities and a reduced global longitudinal strain of –9%.

**Figure 3:**
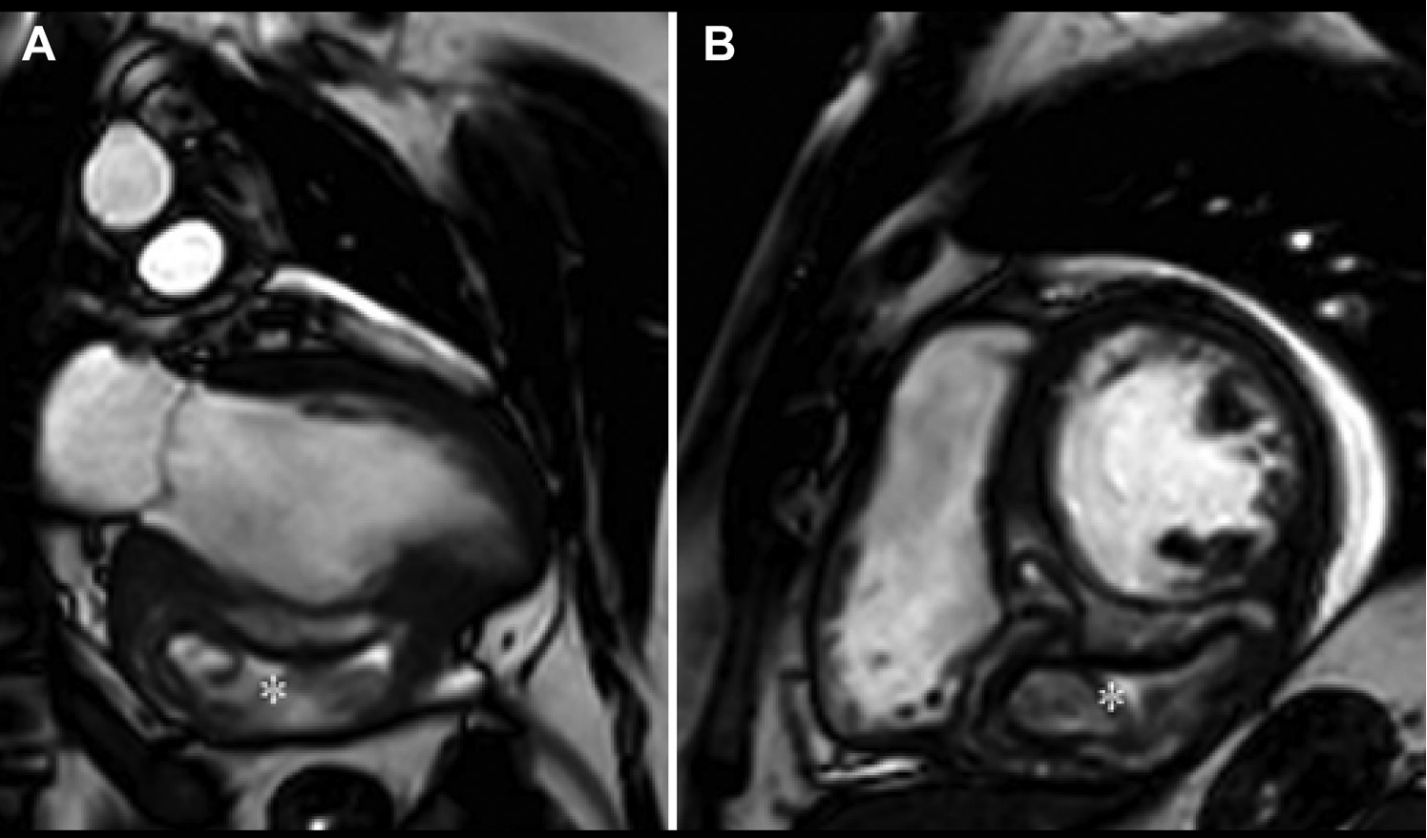
Cardiac magnetic resonance imaging (balanced steady-state free procession) in **(A)** two-chamber view and **(B)** short-axis view showed a multilobulated cystic structure with the appearance of cardiac echinococcosis, partially damaging the myocardium in the inferoseptal region (*asterisks*).

A 38-year-old previously healthy man, originally from Serbia, presented with sudden-onset respiratory-related chest pain to the emergency department of a secondary-level hospital. Elevated cardiac troponin I (315 ng/L, reference <35 ng/L) and C-reactive protein (154 g/L, reference <5 g/L) together with elevated N-terminal prohormone of brain natriuretic peptide (1,510 ng/L, reference <150 ng/L) and an abnormal electrocardiogram (Figure 1) raised suspicion of myocarditis. Next he underwent a transthoracic echocardiogram (TTE), revealing a large mass with a hypoechogenic area in the inferoseptal region of the heart comprising both the left and right ventricles. Left ventricular (LV) ejection fraction (LVEF) was mildly to moderately reduced (40%) due to hypo- and dyskinesia in the affected region, and global longitudinal strain (GLS) was severely reduced (–9.3%; Figure 2, Video 1). There was no shunt flow across the interventricular septum (Video 2) and no concomitant valvular lesions, and the systolic right ventricular function appeared normal. A cMRI examination revealed a 3.5 × 6.5 cm septet hydatid cyst in the inferoseptal region (Figure 3, Videos 3 and 4). Despite the enzyme-linked immunosorbent assay negative serology, cardiac echinococcosis was suspected. The patient was scheduled for surgical excision of the cyst and was transferred to our department. Electrocardiogram-gated CT confirmed what was seen on the cMRI and ruled out communication between the cyst and the left and right ventricles. Furthermore, it showed no significant coronary artery stenoses. Computed tomography over the thoracic and abdominal regions showed no signs of extracardiac involvement. The patient was treated with 200 mg albendazole twice daily for six days before surgery. The patient also received preoperative prophylactic steroids to prevent anaphylactic shock.

**Figure 4:**
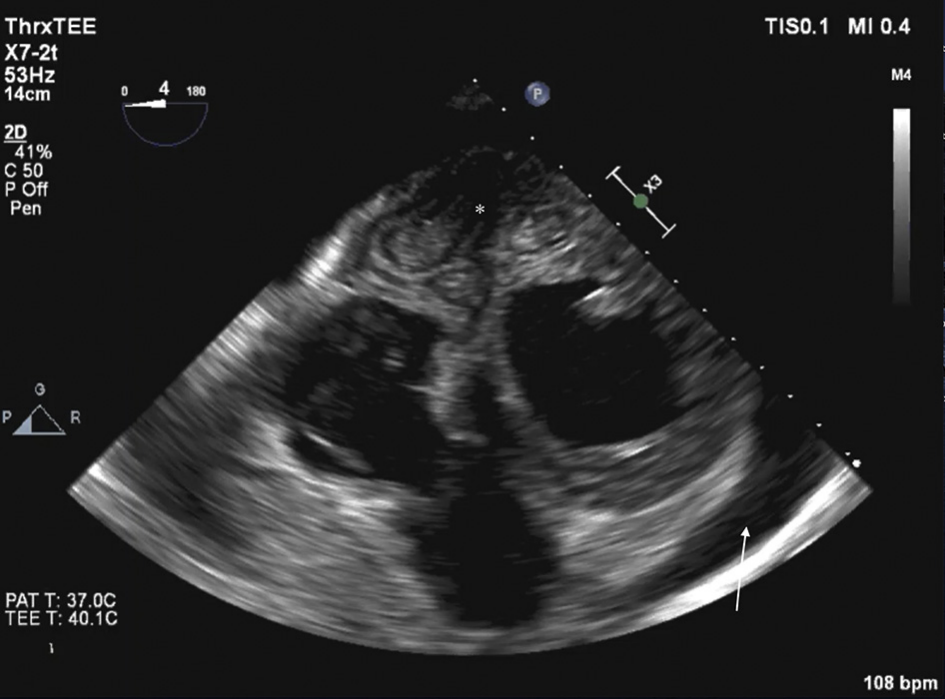
Perioperative TEE transgastric short-axis view revealed cystic structures in the inferoseptal region as well as pericardial effusion.

**Figure 5:**
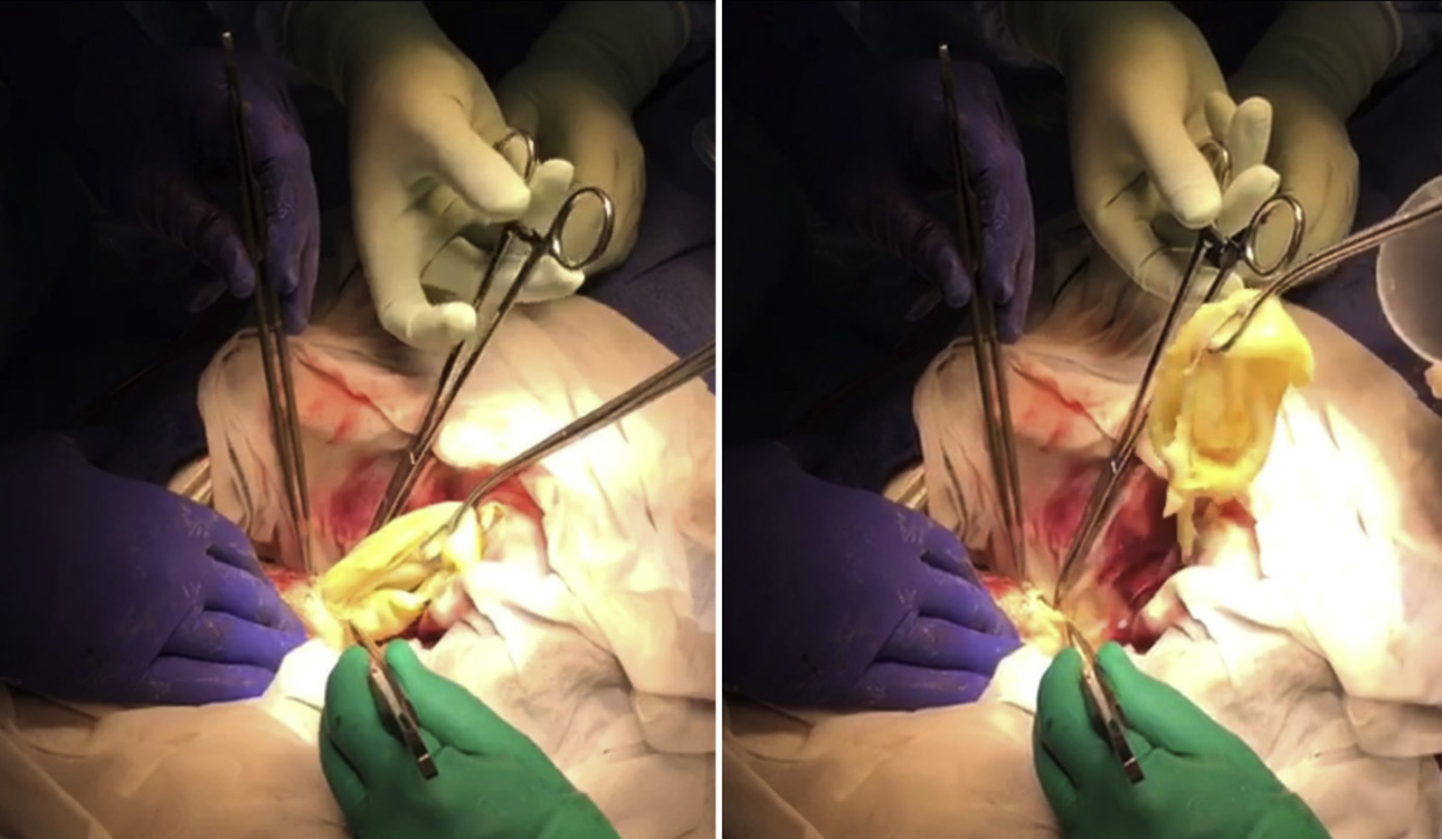
Gross images of the surgical removal of the ruptured large hydatid capsule.

Intraoperative transesophageal echocardiography (TEE) revealed cystic structures in the inferoseptal region and a small amount of pericardial effusion (Figure 4, Video 5). A full median sternotomy was performed, revealing corresponding inflammatory changes in the pericardium and on the epicardial surface. A small amount of pericardial fluid was aspirated, suggesting recent cyst rupture. Cardiopulmonary bypass was initiated, and the heart was arrested by cross-clamping the ascending aorta and administering antegrade cardioplegia, which was iterated every 20 minutes during the cross-clamping. An LV vent was inserted through the right upper pulmonary vein. On full cardiopulmonary bypass, pericardial adhesions were relieved, and the heart was displaced anteriorly in order to inspect the inferiorly situated cyst. Before opening the cyst, a 10% hypertonic saline solution was injected into the cyst and left for 10 minutes. The pericardial surfaces were covered with hydrogen peroxide–soaked towels to prevent direct contact with cyst fluid. The wall of the main cyst together with the inactive daughter cysts was carefully excised to avoid damage of the posterior descending branch of the right coronary artery (Figure 5, Video 6). The cyst cavity was subsequently rinsed with 95% ethanol and 3% hydrogen peroxide solution. A glutaraldehyde-treated bovine pericardial patch was used to reinforce and obliterate the cavity. Weaning from cardiopulmonary bypass was uncomplicated. Before closing the sternotomy, 3% hydrogen peroxide solution was instilled in the pericardium to wash off any remaining parasites. Gore-Tex membrane was used to adapt the pericardium in case the patient should need future cardiac surgery. Intraoperative TEE after removal of the hydatid cysts and obliteration of the cavity with bovine pericardial patches indicated immediate improvement in LV contractility (Video 7).

The postoperative course was completely uneventful. Routine postoperative TTE showed improved myocardial contraction in the previously affected regions and no ventricular septum defect. The patient was discharged to the referral hospital on the seventh postoperative day with a plan to continue albendazole treatment for a total of four weeks postoperatively. The diagnosis of cardiac echinococcosis with *E. granulosus* was later confirmed by the laboratory of the Public Health Agency of Sweden.

The patient continued close follow-up with the cardiologist and infectious disease specialist at the referral hospital. The latest TTE about 12 months postoperatively showed normalization of LVEF (>55%), normal LV dimensions, no signs of regional wall-motion abnormalities, and no valvular heart disease. The GLS was not reported.

## Discussion

Echinococcosis is uncommon in Sweden, with fewer than 35 annual cases since 2005, mostly contracted in high-endemic countries. Cardiac echinococcosis was suspected early due to the TTE and cMRI findings. In line with other reports,^5^ the serology was negative. It has been suggested that ultrasound has better sensitivity for detecting liver echinococcosis than does serological testing, especially when combined with other modalities such as CT and/or magnetic resonance imaging.^5^ This also applies to cardiac echinococcosis, for which both TTE and TEE play important roles in the diagnosis and in planning the surgical management.^6^, ^7^, ^8^ In our patient, a multimodality imaging strategy raised suspicion of cardiac echinococcosis, despite negative serological tests. Rupture of the cyst was suspected as pericardial effusion was present. Transthoracic echocardiography has the advantage of detecting valvular regurgitation or stenosis necessitating concomitant surgical intervention. Global longitudinal strain has previously proven to provide important information regarding regional myocardial function,^9^ but its use in cardiac echinococcosis has not previously been reported. Here, GLS revealed the severely reduced regional function with dyskinesia in the area containing the hydatid cyst.

Cardiac involvement of echinococcosis can have various clinical features, with chest pain and dyspnea being the most common presentations. Congestive heart failure, pericardial tamponade, pulmonary embolism, and superior vena cava syndrome are sometimes observed.^10^ In addition, cyst rupture may cause anaphylactic shock with fatal outcomes if the contents leak into the bloodstream.^11^ Luckily, the hydatid cyst in our patient had no communication with the left or right ventricle. The cyst found in our patient contained very little fluid at the time of surgery and probably ruptured into the pericardium on the day of admission to the emergency department, when the patient presented with sudden-onset respiratory-related chest pain with the characteristics of pericarditis and/or myocarditis. Supporting this is the fact that inflammatory changes and pericardial fluid were found when the pericardium was opened. To our knowledge, this case report is the first description of a nonfatal outcome after cardiac hydatid cyst rupture.

Surgical removal of the cysts associated with cardiac echinococcosis should be considered to prevent potentially fatal cyst rupture, and the results can be excellent.^3^ Complete removal of the capsule is recommended to avoid the risk of recurring disease.^12^ The most appropriate treatment depends on both patient and cyst characteristics.^13^ Although preoperative treatment with albendazole is recommended in liver echinococcosis, no such recommendation for cardiac echinococcosis has been established due to its rare occurrence and because pharmacological therapy has very little effect on hydatid cysts.^3^ Postoperative relapse has been reported,^14^ so postoperative antiparasitic drug therapy should also be considered to minimize that risk. Follow-up with TTE is recommended every three months for at least three years after surgery, but there is no consensus supporting this recommendation.^13^ While human echinococcosis constitutes a major health problem in some parts of the world, and thus is widely recognized by physicians in these endemic areas, it remains uncommon in Sweden and the rest of the Western world. However, with increasing immigration, its occurrence in low-incidence countries continues to grow. Our patient originated from Serbia and grew up in a rural setting with various animals. He regularly returned to his home country to visit his family, when he was likely infected. This emphasizes, besides multimodality imaging, the importance of obtaining a thorough medical history, including questions regarding origin, travel habits, and exposure to livestock farming. This can be managed through multidisciplinary cooperation involving cardiologists, radiologists, infectious disease specialists, and cardiothoracic surgeons.

## Conclusion

Our case demonstrates that a multimodality imaging approach including TTE, cMRI, and CT is important for rapid diagnosis in the workup of cardiac echinococcosis. The presence of pericardial effusion on cardiac imaging should raise suspicion of a ruptured cyst, even in the absence of anaphylactic shock, while the absence of pericardial effusion would argue against rupture into the pericardium. This case also illustrates that GLS supplies important information about regional function in infiltrative myocardial disease such as cardiac echinococcosis.

## Supplementary Data

Video 1 Parasternal short-axis view at the level between the mitral valve and the papillary muscles showing the cystic mass, with hypoechoic regions, in the inferoseptal wall.

Video 2 Parasternal short-axis color Doppler view gave no suspicion of shunt flow across the interventricular septum.

Video 3 Balanced steady-state free procession two-chamber view showing a large hydatid cyst in the inferior wall, affecting LV contractility. A small amount of pericardial effusion can also be seen.

Video 4 Balanced steady-state free procession short-axis view of the hydatid cyst.

Video 5 Perioperative TEE (transgastric short-axis view) showing cystic structures in the inferoseptal region, compromising LV function.

Video 6 Short video from the operating room showing surgical removal of the large hydatid capsule.

Video 7 Perioperative TEE (transgastric short-axis view) after removal of the hydatid cysts and obliteration of the cavity with bovine pericardial patches, resulting in immediate improvement in LV contractility. *post-ECC*, Post extracorporeal circulation.
